# METTL3-mediated m^6^A modification stabilizes TERRA and maintains telomere stability

**DOI:** 10.1093/nar/gkac1027

**Published:** 2022-11-18

**Authors:** Liping Chen, Canfeng Zhang, Wenbin Ma, Junjiu Huang, Yong Zhao, Haiying Liu

**Affiliations:** MOE Key Laboratory of Gene Function and Regulation, State Key Laboratory of Biocontrol, School of Life Sciences, Sun Yat-sen University, Guangzhou 510006, China; The Center for Medical Research, The First People's Hospital of Nanning City, Nanning 530021, China; Scientific Research Center, The Seventh Affiliated Hospital, Sun Yat-Sen University, Shenzhen, 518107, China; MOE Key Laboratory of Gene Function and Regulation, State Key Laboratory of Biocontrol, School of Life Sciences, Sun Yat-sen University, Guangzhou 510006, China; MOE Key Laboratory of Gene Function and Regulation, State Key Laboratory of Biocontrol, School of Life Sciences, Sun Yat-sen University, Guangzhou 510006, China; MOE Key Laboratory of Gene Function and Regulation, State Key Laboratory of Biocontrol, School of Life Sciences, Sun Yat-sen University, Guangzhou 510006, China; MOE Key Laboratory of Gene Function and Regulation, State Key Laboratory of Biocontrol, School of Life Sciences, Sun Yat-sen University, Guangzhou 510006, China

## Abstract

Telomeric repeat-containing RNA (TERRA) is a type of long non-coding RNA transcribed from telomeres, and it forms R-loops by invasion into telomeric DNA. Since either an excessive or inadequate number of R-loops leads to telomere instability, the TERRA levels need to be delicately modulated. In this study, we found that m^6^A modification presents on the subtelomeric regions of TERRA and stabilizes it, and the loss of METTL3 impacts telomere stability. Mechanically, the m^6^A modification on TERRA is catalyzed by METTL3, recognized and stabilized by the m^6^A reader YTHDC1. Knockdown of either METTL3 or YTHDC1 enhances TERRA degradation. The m^6^A-modified TERRA forms R-loops and promotes homologous recombination which is essential for the alternative lengthening of telomeres (ALT) pathway in cancer cells. METTL3 depletion leads to R-loop reduction, telomere shortening and instability. Altogether, these findings reveal that METTL3 protects telomeres by catalyzing m^6^A modification on TERRA, indicating that inhibition or deletion of METTL3 is potentially a new avenue for ALT cancer therapy.

## INTRODUCTION

Telomeres are composed of tandem repeats of the DNA sequence TTAGGG/AATCCC and are located at the physical ends of eukaryotic linear chromosomes, safeguarding the natural DNA ends from being recognized as DNA damage (1–3). It is reported that 85–90% of cancer cells maintain telomere length by telomerase, while the remaining 10–15% elongate telomeres by the alternative lengthening of telomeres (ALT) pathway that utilizes homologous recombination (HR) resulting in telomeric sister chromatin exchange (T-SCE) (4–6). Although the proportion of ALT cancers is lower than that of telomerase-positive cancers, most of the ALT cancers are more malignant and the prognoses are poor. There are lots of special characteristics in ALT cancer cells such as the C-circle, ALT-associated PML bodies (APBs), heterogeneous telomere length, absence of ATRX, telomere recombination and a high telomeric repeat-containing RNA (TERRA) level (7–11). It has been reported that TERRA is required for maintenance of telomere and chromosome stability. Depletion of TERRA transcripts lead to the activation of DNA damage response at chromosome ends, resulting in telomere dysfunction-induced foci (TIFs) (12–14), indicating that TERRA is a potential target for ALT cancer therapy.

TERRA is a type of long non-coding RNA transcribed from subtelomeric regions to telomeres by RNA polymerase II (Pol II), with heterogeneous length ranging from 100 bp to 9 kb (8,9). It has been reported that TERRA can bind to telomeres and form R-loops (the three-stranded nucleic acid structures that consist of an RNA–DNA hybrid and a displaced DNA strand) both in yeast and in human cells (15–20). The R-loops formed by TERRA may act as a double-edged sword in telomere length maintenance (21). Rajika Arora *et al.* revealed that depletion of RNase HI results in telomeric R-loop accumulation, which induces replication stress and telomere shortening (20). In contrast, overexpression of RNase HI causes a decrease in HR and telomere shortening in recombination-competent yeast and ALT cells, indicating that TERRA-formed R-loops play a physiological role at telomeres (16,20). The double-faced roles of TERRA in telomere length maintenance raises the hypothesis that TERRA expression should be tightly regulated to maintain appropriate levels required for ALT.

*N* ^6^-Methyladenosine (m^6^A) is one of the most prevalent and reversible internal RNA modifications among numerous post-transcriptional modifications identified in eukaryotic mRNAs and non-coding RNAs (22). It occurs at the consensus motif RRACH (R is G, A or U; H is U, A or C) and regulates RNA transcription, splicing, degradation and translation (22–29). The m^6^A modification of RNAs is catalyzed by its writers, the m^6^A methyltransferase enzyme complex, including METTL3, METTL14 and WTAP (30–32), and it is removed by the m^6^A demethylase enzymes such as fat mass obesity-associated protein (FTO) and Alk B homolog 5 (ALKBH5) (33,34). The m^6^A modification is recognized by several reader proteins, including the YTH domain family (YTHDF1, YTHDF2 and YTHDF3), YTH domain-containing proteins (YTHDC1 and YTHDC2) and IGF2BPs (IGF2BP1, IGF2BP2 and IGF2BP3). The readers bind to m^6^A methylation sites and regulate RNA stability, nuclear export, splicing and translation (27,35–39).

Previously, we revealed that m^6^A-modified RNAs regulate repair of double-strand DNA breaks (DSBs) (40). Briefly, they form RNA–DNA hybrids at DSB sites and then recruit RAD51 and BRCA1 to the DSB sites, promoting HR repair and maintaining genome stability (40). Whether and how METTL3-m^6^A plays a role in maintaining telomere stability in ALT cells are largely unknown. Here, we report that METTL3 and YTHDC1 catalyze and recognize m^6^A modification on TERRA subtelomeric regions, respectively, and regulate TERRA stability. Then m^6^A-modified TERRA forms R-loops at telomeres, regulating telomeric HR, and the loss of METTL3 leads to telomere instability in ALT cells.

## MATERIALS AND METHODS

### Cell culture

U2OS, VA13, CAL27 and HEK293T cells were obtained from the American Type Culture Collection (Manassas, VA, USA) and were cultured at 37°C and 5% CO_2_. U2OS cells were grown in Dulbecco’s modified Eagle’s medium (DMEM; Hyclone) with 10% newborn calf serum (PAA Laboratories) and 100 U/ml penicillin/streptomycin (Hyclone). VA13, CAL27 and HEK293T cells were grown in DMEM (Hyclone) with 10% fetal bovine serum (FBS; Gibco) and 100 U/ml penicillin/streptomycin. All cells were negative for mycoplasma contamination.

### Gene silencing and overexpression

METTL3, ALKBH5 and RNase HI were deleted by small interfering RNA (siRNA) using Lipofectamine RNAiMAX Transfection Reagent (Invitrogen) according to the manufacturer's instructions. The sequences of siRNAs are as follows: siMETTL3-1, 5′-CUGCAAGUAUGUUCACUAUGAdTdT-3′; siMETTL3-2, 5′-GCACUUGGAUCUACGGAAUCCdTdT-3′; siALKBH5-1, 5′-ACAAGUACUUCUUCGGCGUdTdT-3′; siALKBH5-2, 5′-GCGCCGUCAUCAACGACUAdTdT-3′; siRNase HI, 5′-ACCAAAGAGCGGAAAUUCAUGdTdT-3′; and negative control siRNA (NC), 5′-UUCUCCGAACGUGUCACGUTTdTdT-3′.

METTL3 and YTHDC1 were also knocked down by stable expression of short hairpin RNA (shRNA). Lentivirus with shDNA was packaged in HEK293T cells using calcium chloride transfection. Viruses were collected and used for infection of U2OS, VA13 or CAL27 cells. Cells were selected with puromycin to obtain a mixed population of clones and they were kept continuously in culture for the execution of the experiments. The sequences of shDNAs are as follows: shMETTL3-1, 5′-CCCACACAATGTGCAACCCAACTAGTGAAGCCACAGATGTAGTTGGGTTGCACATTGTGTGGT-3′; shMETTL3-2, 5′-ATCAGTGGATCTGTTGTGATATTAGTGAAGCCACAGATGTAATATCACAACAGATCCACTGAG-3′; shYTHDC1-1, 5′-GATCGACAGGAAATTGAACTTGATTCAAGAGA-3′; and shYTHDC1-2, 5′-GATCGAAGTGGATAGACGTGCAATTCAAGAG-3′.

For plasmid transient transfection, polyethyleneimine (PEI; Yeasen) was used for HEK293T cells, while Lipofectamine 3000 (Invitrogen) was used for U2OS and CAL27 cells, according to the manufacturer's instructions.

### Plasmid construction

THe ALKBH5 gene was amplified from HEK293T mRNAs and cloned into HA-dCasRx-2A-EGFP (referred to as ‘dCas13-ALKBH5’ in the text and figures) (a gift from the Zhou Songyang lab). The YTHDC1 gene was amplified from HEK293T mRNAs and cloned into pLenti-HA/Flag or HA-dCasRx-2A-EGFP (referred to as ‘dCas13-YTHDC1’ in the text and figures). The small guide RNAs (sgRNAs) targeted to the 15q-TERRA subtelomere or scrambled were synthesized and cloned into the CasRx gRNA cloning backbone (a gift from the Zhou Songyang lab). The sequences of sgRNAs are as follows: sg-15q-1, 5′-CCGCCCGCCCGGGTCTGACCTG-3′; sg-15q-2, 5′-CGCAGTGGCCCCAACGTCTGTG-3′; sg-15q-3, 5′- TTACGGTGACCCCCAGGTCT-3′; and scrambled sequence, 5′-TCACCAGAAGCGTACCATACTC-3′. The full length of the 15q-TERRA subtelomeric region was amplified from HEK293T genomic DNA and cloned downstream of the Renilla luciferase gene in the psiCHECK-2 vector (Promega) (a gift from the Jun Cui lab). The m^6^A consensus sequence mutants (A to T mutation) were generated by site-directed mutation based on wild-type 15q-TERRA. The RNase HI D210N mutant was generated by site-directed mutagenesis of the wild-type RNase HI gene and cloned into pLVX-AcGFP1-N1 (Clontech).

### Construction of the m^6^A-editing system and the YTHDC1-binding system based on dCas13

For the m^6^A-editing system, U2OS cells were co-transfected with 15q-TERRA targeting sgRNAs (sg-15q-1 or sg-15q-2) and plasmid dCas13-ALKBH5-WT or dCas13-ALKBH5-MUT using Lipofectamine 3000. At 72 h post-transfection, cells were collected and RNA immunoprecipitation (RIP)-quantitative polymerase chain reaction (qPCR), m^6^A-modified RIP (MeRIP)-qPCR and reverse transcription (RT)–qPCR were performed.

For the YTHDC1 binding system, METTL3-depleted (shMETTL3) or control U2OS cells were co-transfected with dCas13-YTHDC1 and 15q-TERRA targeting sgRNAs (sg-15q-1 and sg-15q-3) using Lipofectamine 3000. At 72 h post-transfection, cells were collected and RIP-qPCR and RT–qPCR were performed.

### TERRA RNA pull-down

RNA pull-down assay was performed as described previously (41). A 100 μg aliquot of total RNAs extracted from METTL3-deficient and control U2OS cells was treated with DNase I at 37°C for 1 h to degrade DNA. Subsequently, 2.5 mM EDTA (pH 8.0) was added to stop the reaction. After adding in twice the volume of hybridization buffer [500 mM NaCl, 1% sodium dodecyl sulfate (SDS), 100 mM Tris–HCl 7.0, 10 mM EDTA (pH 8.0), 15% formamide and 1 mM dithiothreitol (DTT)], the RNAs were incubated with 100 pmol biotin-labeled telomeric C probe [5′-biotin-(CCCTAA)_3.5_] in a 3D rotator at 37°C for 4 h; the biotin-labeled telomeric G probe [5′-biotin-(TTAGGG)_3.5_] was used as negative control. Streptavidin magnetic beads (Z5481, Promega) were washed with hybridization buffer three times, blocked with 1 mg/ml bovine serum albumin (BSA) for 1 h at room temperature, washed again three times and suspended in an equal volume of hybridization buffer. The RNAs hybridized with biotin probes were incubated with beads for 1 h at 37°C. Beads:biotin probes:RNA adducts were captured by magnets and washed six times with wash buffer (2× SSC, 0.5% SDS and 1 mM DTT) and twice in TE buffer. The RNAs were eluted with 200 U of DNase I, followed by Trizol (R480201, Magen Biotechnology) extraction. The products were detected by slot-blot using ^32^P-labeled telomeric C probe for TERRA detection and anti-m^6^A antibody (1:1000 dilution, 202003, Synaptic Systems) for m^6^A detection.

For assay to detect which region of TERRA is modified by m^6^A, the beads:biotin probes:RNA adducts were blocked by telomeric C probe and then digested in reaction buffer [20 mM Tris–HCl (pH 7.8), 40 mM KCl, 8 mM MgCl_2_ and 1 mM DTT] by RNase H (M0297L, New England BioLabs) or RNase A (2158, Takara), respectively. Buffer-only reaction was used as control. After digestion, the supernatant of the RNase H-treated sample, beads of the RNase A-treated sample and all components of the control group were extracted by Trizol. DNase I-containing recombinant RNase inhibitor (RRI) (2313A, Takara) was used to remove the blocking probes. Finally, slot-blot was performed to detect m^6^A and TERRA.

### m^6^A-modified RNA immunoprecipitation

Total RNAs were extracted from METTL3-deficient and control U2OS cells. DNAs were removed from total RNAs by DNase I at 37°C for 1 h. Immunoprecipitation of m^6^A-modified RNAs was performed as previously described (42) using primary antibody against m^6^A (1:100 dilution). For METTL3-deficient U2OS cells, TERRA in the products was detected by slot-blot using ^32^P-labeled telomeric C probe. For the dCas13–ALKBH5 system, the precipitated TERRA was detected by RT–qPCR.

### RNA half-life assay and qPCR

U2OS cells with METTL3 or YTHDC1 stably knocked down were treated with 5 μg/ml actinomycin D (AcTD; A1410, Sigma-Aldrich) for 0, 0.5, 1, 2 or 4 h. Total RNAs were extracted by Trizol extraction, and reverse-transcribed using the PrimeScript RT reagent kit (AU311, TransGen Biotech) with random primers and TERRA-specific primers (RT-C×5, 5′-CCCTAACCCTAACCCTAACCCTAACCCTAA-3′; RT-C×3, 5′-CCCTAACCCTAACCCTAA-3′). cDNA was used for real-time PCR using 2× qPCR mixture (KTSM1401S, KT HEALTH). 18S RNA was used as internal control. The PCR primer sequences are shown in Supplementary Table S1.

### Chromatin immunoprecipitation (ChIP)

METTL3-depleted (shMETTL3) or control U2OS cells were cross-linked by 1% formaldehyde and subjected to ChIP assays performed as described previously (43). For Pol II-ChIP, the antibodies used were as follows: anti-RNA polymerase II CTD repeat YSPTSPS (phosphor S2) (1:100 dilution, ab5095, Abcam) or anti-IgG (D110502, Sangon Biotech). ^32^P-Labeled telomeric G probe was used to detect the telomere signal in products by slot-blot.

For R-ChIP, green fluorescence protein (GFP)–antibody conjugated beads (KTSM1334, KT Health) were used. After treatment with DNase I for 1 h at 37°C, products were detected by slot-blot using ^32^P-labeled telomeric C probe.

For YTHDC1-ChIP, Flag-antibody conjugated beads (HY-K0207-1mL, MCE) were used. ^32^P-Labeled telomeric G probe was used to detect the telomere signal in products by slot-blot.

### *In vitro* RNA stability assay

METTL3-deficient or control U2OS cells were collected and lysed in RNase-free lysis buffer [1% NP-40, 0.25% sodium deoxycholate, 50 mM Tris–HCl (pH 7.4), 150 mM NaCl, 1 mM EDTA (pH 8.0), 1 mM phenylmethylsulfonyl fluoride (PMSF)] on ice for 10 min. After centrifugation at 14 000 *g* for 20 min at 4°C, the supernatant was incubated with 0.5 μg of synthetic biotin-labeled 15q-TERRA (General Biol) with or without m^6^A modification at 37°C for 0 or 2 h. The sequence of 15q-TERRA is as follows, with the ‘A’ in parentheses modified or not as indicated: Biotin-15q-TERRA, 5′-biotin-CUCCUCAGGUCAG(A)CCCGGGCGGGCGGGCUGAGGGUACCGCGAGGGCGGAG-3′. After denaturation at 94°C for 5 min, samples were separated by urea-polyacrylamide gel electrophoresis (PAGE), transferred to an Amersham Hybond-N^+^ membrane (GE Healthcare) and detected with horseradish peroxidase (HRP)-conjugated streptavidin (1:3000 dilution, FXP026-100, 4Abio). After chemiluminescence visualization, the membrane was incubated with m^6^A antibody (1:1000 dilution) and fluorescein-conjugated IRDye-680CW goat anti-rabit (926–32220, LI-COR Biosciences) to detect m^6^A-modified TERRA.

### RNA immunoprecipitation (RIP)

In the RIP assay with ALKBH5 or YTHDC1 targeted to 15q-TERRA, cells were cross-linked by 1% formaldehyde and subjected to RIP using hemagglutinin (HA)-antibody-conjugated beads (HY-K0201-1ml, MCE) as described previously (44). The amount of 15q-TERRA co-immunoprecipitated with HA-dCas13-ALKBH5 was detected by RT–qPCR and Flag-YTHDC1 was assessed by slot-blot. The pull-down efficiency was calculated as ‘percentage of input’, and the percentage was then normalized to cells transfected with scramble sgRNA; the resulting ‘fold’ is equal to the relative amount of TERRA.

In the RIP assay with Flag-YTHDC1, the same experiment as above was performed by using Flag-antibody-conjugated beads instead. The TERRA amount was detected by RT–qPCR.

### Luciferase and reporter assay

U2OS cells were plated in a 24-well plate. After knocking down METTL3 with siRNA, the cells were transfected using Lipofectamine 3000 with psiCHECK-2 vector expressing wild-type or the m^6^A consensus sequence mutant (A to T mutation) 15q-TERRA subtelomeric region. Cells were collected and luciferase activity was measured with the dual-luciferase assay with a Luminoskan Ascent luminometer (Thermo Fisher Scientific) according to the manufacturer's protocol. Gene expression reporter activity was determined by normalization of the Renilla luciferase signal to the firefly luciferase signal.

### Immunofluorescence-fluorescent *in situ* hybridization (IF-FISH)

Cells with METTL3 stably knocked down were grown on coverslips and fixed in 4% paraformaldehyde for 15 min at room temperature, then permeabilized with 0.5% Triton X-100. After blocking with 5% goat serum, cells were incubated overnight at 4°C with primary antibodies against γH2AX (1:400 dilution, 2577, Cell Signaling Technology), RPA1 (1:100 dilution, sc-28304, Santa Cruz), RAD51 (1:100 dilution, sc-8349, Santa Cruz) or S9.6 (1:100 dilution, MABE1095, Millipore) for 1.5 h at room temperature. After washing with phosphate-buffered saline–Tween-20 (PBST), cells were incubated with DyLight 488- or 555-conjugated secondary antibody for 1.5 h at room temperature. After washing with PBST, the cells were fixed with 4% paraformaldehyde again for 30 min at room temperature. Then cells were dehydrated using ethanol series solutions, denatured at 85°C for 5 min and hybridized with fluorescein isothiocyanate (FITC)-labeled peptide nucleic acid (PNA) telomeric C probe (Panagene, Daejeon) or Cy3-labeled PNA telomeric G probe (Panagene, Daejeon) for at least 2 h at 37°C. Finally, cells were washed and mounted with 4′,6-diamidino-2-phenylindole (DAPI) and visualized using a Zeiss microscope.

### IF-RNA FISH

Firstly, native FISH was performed with cells grown on coverslips. Cells were fixed in 4% paraformaldehyde for 15 min at room temperature, then permeabilized with 0.5% Triton X-100 for 30 min also at room temperature. Hybridizations were performed by using FITC-labeled PNA telomeric C probe for 3 h at 37°C, then IF was performed. Cells were fixed with 4% paraformaldehyde again for 30 min at room temperature. After blocking with 5% goat serum, cells were incubated for 1.5 h at room temperature with primary antibodies against TRF1 (1:100 dilution, GTX77605, GeneTex). After washing with PBST, cells were incubated with DyLight 555-conjugated secondary antibody for 1.5 h at room temperature. Finally, cells were washed and mounted with DAPI and visualized using a Zeiss microscope or Leica microscope.

To confirm that TERRA but not telomere DNA was detected in the native FISH, additional native FISH with RNAs which had been digested was performed. Briefly, cells were permeabilized with 0.5% Tween-20 for 10 min at room temperature, and then incubated with 100 μg/ml RNase A and 10 U of RNase T1 (EN0541, Thermo Fisher Scientific) or with PBS for 20 min at 37°C. Subsequently, cells were fixed, permeabilized and hybridized in the same way as described above.

### Chromosome orientation FISH (CO-FISH)

The U2OS cells were treated with 1 μg/ml bromodeoxyuridine (BrdU; 100166, MP Biomedicals) for 20 h, and 1 μg/ml colchicine was added during the last 5 h to arrest cells at metaphase. Cells were collected and then hypotonically treated with 75 mM KCl solution for 30 min at 37°C and fixed with methanol:acetic acid (3:1) twice. Cells were spread onto clean cold slides, and digested with 1 mg/ml pepsin for 30 s at 37°C. After incubation with Hoechst 33258 (H1398, Invitrogen) for 30 min at room temperature, the slides were exposed to 365 nm UV light (UVP-CL1000) for 40 min. Then samples were digested with 2 U/μl exonuclease III (M1815, Promega) for 2 h at 37°C and hybridized with FITC-labeled PNA C probe and Cy3-labeled PNA G probe. Chromosome were stained with DAPI and visualized using a Leica microscope.

### Quantitative FISH (q-FISH)

Cells were treated with 1 μg/ml colchicine for 6 h. Metaphase-enriched cells were hypotonically treated, fixed and spread onto slides as in the CO-FISH protocol. Telomeres were hybridized with FITC-labeled PNA C probe (for U2OS cells) or Cy3-labeled PNA G probe (for VA13 cells) after denaturation at 85°C. Chromosome were stained with DAPI and visualized using a Zeiss microscope.

### Telomere restriction fragment (TRF)

Mean telomere length was evaluated by TRF analysis as described previously (45). DNAs were isolated from METTL3-deficient or control U2OS cells using phenol–chloroform. A 3 μg aliquot of DNAs was digested with RsaI (R0167L, New England BioLabs), HinfI (R0155L, New England BioLabs) and RNase A, and separated on a 0.7% agarose gel. After being dried and denatured, the agarose gel was hybridized with ^32^P-labeled telomeric G probe and exposed to a PhosphorImager screen.

### Cell viability

METTL3-depleted (shMETTL3) or control U2OS cells (2 × 10^3^) or CAL27 cells (1 × 10^3^) were plated on a 96-well plate. After the cells had adhered, cell viability was measured with Cell Counting Kit-8 (B34302, Bimake) following the protocol provided by the manufacturer. Cell viability assays were performed every day for six consecutive days.

### Colony formation assays

METTL3-depleted (shMETTL3) or control U2OS cells were plated into 6-well plates at low seeding density (5000/well). Ten days later, colonies were stained with crystal violet (C0121, Beyotime) and counted using ImageJ.

### Immunoblotting

Cells were lysed in SDS lysis buffer [50 mM Tris–HCl (pH 6.8), 2% SDS, 0.1% bromophenol blue, 10% glycerol, 1% β-mercaptoethanol] and boiled for 10 min. Then proteins were separated on an SDS–PAGE gel and transferred to a polyvinylidene difluoride membrane (PALL). The following antibodies were used for immunoblotting: anti-METTL3 (1:2000, ab195352, Abcam), anti-YTHDC1 (1:2000, ab122340, Abcam), anti-histone H3 (1:1000, ab1791, Abcam), anti-TRF1 (1:1000 dilution), anti-TRF2 (1:2000 dilution, 05-513, Merck), anti-POT1 (1:2000 dilution, NB500-176, Novus Biologicals), anti-RNase HI (1:2000, 15606–1-AP, Proteintech), anti-glyceraldehydephosphate dehydrogenase (GAPDH; 1:5000, 60004–1-Ig, Proteintech), anti-α-tubulin (1:5000, 66031–1-Ig, Proteintech), HRP-conjugated goat anti-rabbit (KPL, Inc.) or goat anti-mouse (KPL, Inc.).

### Experimental repeats and statistical analysis

In this study, all experiments have been performed with at least three independent biological repeats. For all of the statistical charts, values are presented as means ± standard error of the mean (SEM) of all biological repeats. For foci analysis by microscopy, >100 cells were analyzed in each biological repeat. For analysis of telomere ends, the numbers of quantified telomeres were specified in the figure legends.

Statistical analysis was performed using GraphPad Prism version 8. Two-tailed unpaired Student's *t*-test was used for comparisons between two samples. *P*-values <0.05 were considered statistically significant.

## RESULTS

### m^6^A modification presents on the subtelomeric regions of TERRA

**Figure 1.**
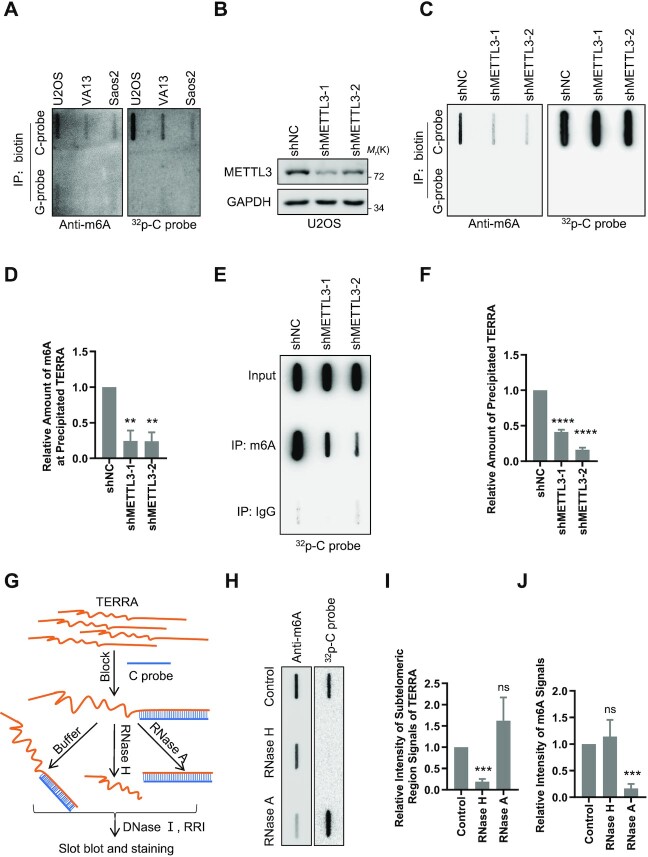
m^6^A modification is present on the subtelomeric regions of TERRA. (**A**) m^6^A signals were detected on TERRA. TERRA was pulled down by biotin-labeled C probe, and biotin-labeled G probe was used as the negative control. Slot-blot was performed to detect m^6^A with anti-m^6^A antibody in the indicated ALT cell lines. Total TERRA was detected by ^32^P-C probe as loading control. (**B**) Western blot analysis of METTL3 knockdown efficiency in U2OS cells. (**C**) TERRA m^6^A signals decrease after METTL3 knockdown. METTL3 was depleted in U2OS cells, and m^6^A modification was detected as in (A). (**D**) Quantification of (C). The relative intensity of precipitated m^6^A signals. (**E**) TERRA was pulled down by m^6^A antibody. MeRIP assay was performed in METTL3-depleted and control U2OS cells using anti-m^6^A antibody. TERRA in precipitates was detected by slot-blot using ^32^P-C probe. The same amount of TERRA was used for MeRIP assay. (**F**) Quantification of (E). The relative intensity of precipitated TERRA signals. (**G–J**) m^6^A localizes at subtelomeric regions of TERRA. (G) Schematic diagram. The telomeric region of TERRA was blocked by the C probe and then digested by RNase H or RNase A, respectively. The buffer without enzyme was used as control. After digestion, DNase I-containing recombinant RNase inhibitor (RRI) was used to remove the blocking probes. Finally, slot-blot was performed to detected m^6^A and TERRA signals (H). (I) Quantification of (H). The relative intensity of subtelomeric region signals of TERRA. (J) Quantification of (H). The relative intensity of m^6^A signals. Note: all values are means ± SEM of at least three independent experiments. Two-tailed unpaired Student's *t*-test was used to determine the statistical significance (**P <0.01, ***P <0.001, ****P <0.0001). For details, please refer to ‘Experimental repeats and statistical analysis’ in the Materials and Methods.

To find out whether there is m^6^A modification on TERRA, we pulled down TERRA using biotin-labeled telomeric C probe [5′-biotin-(CCCTAA)_3.5_] in different ALT cell lines, including U2OS, VA13 and Saos2 cells. The m^6^A modification on TERRA was detected by slot-blot using m^6^A antibody. Biotin-labeled telomeric G probe [5′-biotin-(TTAGGG)_3.5_] was used as the negative control in the pulldown assay. We found that the m^6^A signals exist on TERRA pulled down by the telomeric C probe but not in the control group (Figure 1A). Next, we knocked down the main m^6^A methyltransferase METTL3 in the U2OS cells and repeated the TERRA pulldown assay. The results showed that deletion of METTL3 leads to a significant decrease of m^6^A modification on TERRA as the same amount of pulled down TERRA was loaded (Figure 1B–D). Furthermore, we performed m^6^A RNA IP (MeRIP) using m^6^A antibody to pull down m^6^A-modified TERRA. The results showed that TERRA is pulled down by m^6^A antibody, and loss of METTL3 decreases the amount of precipitated TERRA (Figure 1E, F). The above results suggest that there is m^6^A modification on TERRA, and METTL3 is responsible for this modification.

Since TERRA is transcribed from subtelomeric regions into the telomeric tract, TERRA contains both subtelomeric and telomeric regions (8,9). To explore which region of TERRA is modified by m^6^A, a telomeric C probe was added to form RNA–DNA hybrids with the telomeric regions of TERRA while the subtelomeric regions remain single stranded. Then RNase H or RNase A was added to degrade RNA–DNA hybrids or single-stranded RNAs, respectively (Figure 1G). The remaining RNAs were detected by slot-blot and stained with m^6^A antibody and telomeric C probe after DNAs were digested (Figure 1G). The results showed that the m^6^A signal is present after RNase H digestion but not after RNase A digestion, which is in contrast to the telomere signal (Figure 1H–J), suggesting that m^6^A modification mainly presents on the subtelomeric regions of TERRA.

### m^6^A modification regulates TERRA stability

**Figure 2.**
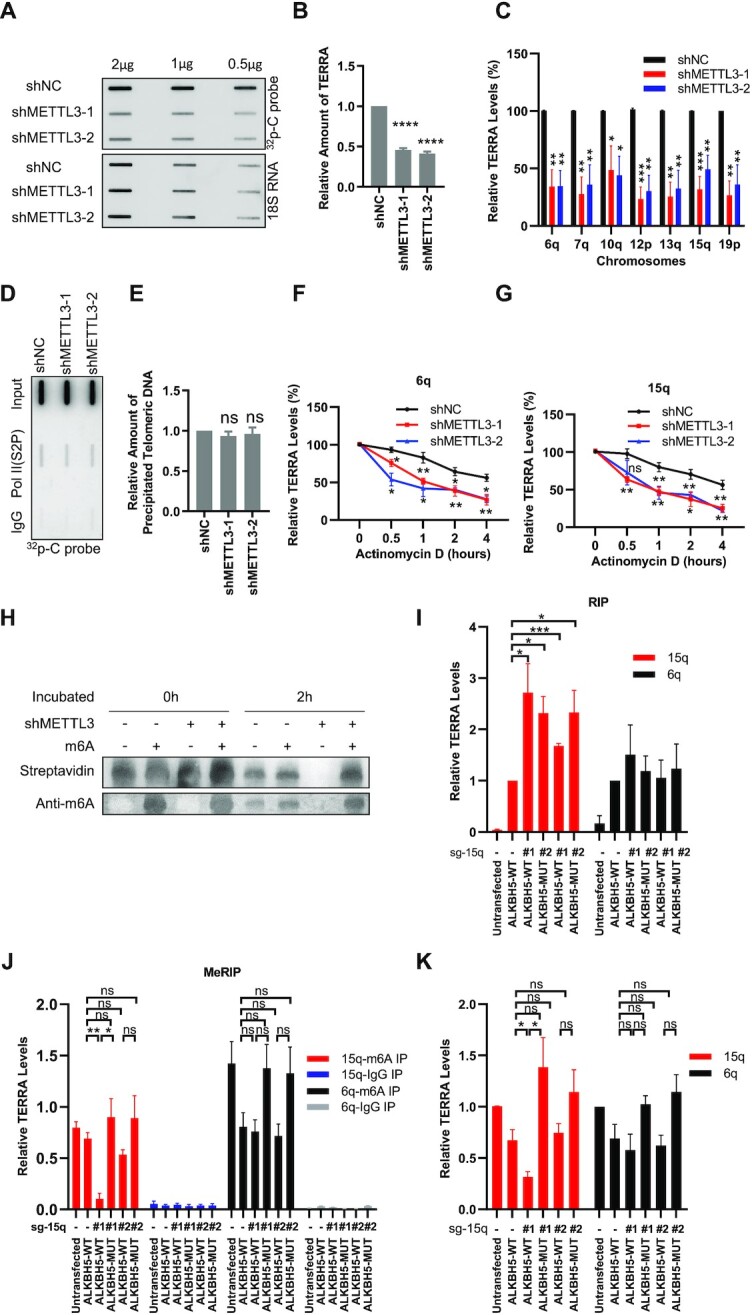
METTL3-mediated m^6^A modification regulates TERRA stability. (**A**) TERRA decreases in METTL3-depleted U2OS cells. Slot-blot was performed to determine the TERRA level, with 18S RNA as loading control. (**B**) Quantification of (A). The amount of TERRA was calculated as TERRA intensity/18S RNA intensity, and then normalized to the shNC group. (**C**) The TERRAs on different chromosomes in METTL3-depleted U2OS cells were detected by RT–qPCR. The 18S RNAs were used for normalization. (**D**) Active Pol II on telomeres does not change in METTL3-depleted U2OS cells. ChIP was performed using Ser2-phosphorylated Pol II (Pol II S2P) antibody, and telomeres were detected using ^32^P-C probe by slot-blot. (**E**) Quantification of (D). The relative intensity of precipitated telomeric DNA signals. (**F, G**) TERRA degrades rapidly in METTL3-depleted U2OS cells. Transcription was inhibited by 5 μg/ml AcTD in METTL3-depleted and control U2OS cells. The TERRA levels of 6q chromosome (F) and 15q chromosome (G) were determined by RT–qPCR at the indicated time points. The 18S RNAs were used for normalization. (**H**) The m^6^A-modified 15q-TERRA fragment is more stable than the unmodified fragment. The biotin-15q-TERRA fragments were incubated with METTL3-depleted or control U2OS cell lysate for 0 or 2 h. The remaining RNA levels were analyzed by urea-PAGE and stained with streptavidin. The m^6^A signals were detected with m^6^A antibody. (**I**) dCas13–ALKBH5 binds to 15q-TERRA. Wild-type and mutated dCas13–ALKBH5 were artificially guided to 15q-TERRA by sg-15q-1 or sg-15q-2 in U2OS cells. RIP was performed by pulling down dCas13–ALKBH5. TERRA was detected by RT–qPCR. The 18S RNAs were used for normalization. (**J**) 15q-TERRA m^6^A modification decreased with dCas13–ALKBH5-WT targeting to the first RRACH motif. U2OS cells were co-transfected with plasmid expressing dCas13–ALKBH5-WT or dCas13–ALKBH5-MUT and sgRNAs targeting 15q-TERRA (sg-15q-1 or sg-15q-2). MeRIP with m^6^A antibody was performed. TERRA was detected by RT–qPCR. (**K**) dCas13–ALKBH5-WT targeted to the first RRACH motif reduces the 15q-TERRA level in U2OS cells. Cells were transfected as in (J), 15q- and 6q-TERRA were detected by RT–qPCR without MeRIP. The 18S RNAs were used for normalization. (*P <0.05, **P <0.01, ***P <0.001, ****P <0.0001).

It has been reported that m^6^A modification can regulate RNA metabolism, including transcription, splicing and stability, thus altering a series of cell biological behaviors (38). To explore the function of m^6^A modification on TERRA, we detected the TERRA levels in METTL3-deficient U2OS cells by slot-blot assay. The results showed that knockdown of METTL3 leads to significant reduction of total TERRA levels (Figure 2A, B), with 18S RNA as loading control, which does not change after METTL3 knockdown (Supplementary Figure S1A, B). It is well known that TERRA is transcribed from multiple chromosome ends (8,9). Therefore, we randomly chose TERRA transcribed from seven different chromosome ends (6q, 7q, 10q, 12p, 13q, 15q and 19p), and detected the expression levels by RT–qPCR using primers targeting subtelomeric sequences. The results showed that individual TERRA molecules are also significantly reduced after METTL3 depletion (Figure 2C).

To find out how METTL3 impacts TERRA levels, we first studied whether the TERRA transcription by Pol II was affected. ChIP was performed to detect Pol II binding with telomeres using the antibody against Ser2-phosphorylated Pol II (Pol II S2P). The results showed that METTL3 knockdown does not affect the binding of Pol II to telomeres (Figure 2D, E), suggesting that METTL3 does not regulate TERRA transcription. Hence, we moved on to check whether m^6^A modification regulates TERRA stability, and found that TERRA transcribed from chromosomes 6q and 15q displays a shorter half-life in METTL3 knockdown cells than in control cells (Figure 2F, G), raising the possibility that reduced m^6^A modification on TERRA leads to its shortened half-life.

To test this possibility, we searched for the m^6^A conserved motif RRACH in the subtelomeric regions of the TERRA in Figure 2C from transcription start sites as described previously (46–48). The results showed that all of these TERRA subtelomeric sequences contain several or many RRACH motifs (Supplementary Figure S1C). To evaluate the effect of m^6^A modification on TERRA stability, we chose the subtelomeric region of 15q-TERRA for further study, whose half-life decreased significantly after METTL3 depletion (Figure 2G). The 451 bp subtelomeric region of 15q-TERRA, including four RRACH motifs (Supplementary Figure S1C), was cloned downstream of Renilla luciferase with each adenine mutated into thymine (TA-1, TA-2, TA-3 and TA-4) (Supplementary Figure S1D). The luciferase activity in NC cells decreases significantly in TA-1 but not in TA-2, TA-3 and TA-4 mutation groups as compared with the wild-type group (Supplementary Figure S1E). In addition, the TA-1 mutation group was not affected by METTL3 knockdown, whereas the luciferase activity in TA-2, TA-3 and TA-4 groups decreased significantly (Supplementary Figure S1E). These results indicate that the first m^6^A consensus sequence may be the key site for m^6^A modification and regulates 15q-TERRA stability.

Next, we synthesized 51 nt subtelomeric fragments of 15q-TERRA with or without m^6^A modification at the first m^6^A consensus sequence (Supplementary Figure S1F), and incubated it with the cell lysate, which contains nuclear factors (Supplementary Figure S1G). We observed that after treatment with METTL3-deficient cell lysate, 15q-TERRA fragments without m^6^A modification degrade within 2 h, whereas 15q-TERRA fragments with m^6^A modification are not degraded (Figure 2H, lanes 3, 4, 7 and 8). However, in the samples treated with control cell lysate, unmodified exogenous 15q-TERRA fragments are retained due to m^6^A modification by endogenous METTL3 during incubation (Figure 2H, lanes 1, 2, 5 and 6).

To further confirm that the attachment of a methyl group at the first m^6^A site is responsible for maintenance of TERRA stability, we artificially removed or did not remove the m^6^A modification at the first site by using the CRISPR–dCas13 system, in which the Cas13 is endonuclease inactive and fused with wild-type or demethylase inactivation-mutated ALKBH5, respectively. ALKBH5 is an RNA demethylase and regulates TERRA stability (Supplementary Figure S1H, I). The dCas13–ALKBH5 RIP assay showed that dCas13–ALKBH5-WT and dCas13–ALKBH5-MUT are successfully targeted to the 15q-TERRA but not the 6q-TERRA by sg-15q-1 and sg-15q-2 (Figure 2I; Supplementary Figure S1J). The m^6^A modification on 15q-TERRA is significantly decreased by dCas13–ALKBH5-WT combined with sg-15q-1, which was targeted to the first RRACH motif, but not with sg-15q-2, whose targeting site is 98 bp away (Figure 2J; Supplementary Figure S1K). Also, the 15q-TERRA level decreases significantly only in cells co-transfected with dCas13–ALKBH5-WT and sg-15q-1 (Figure 2K). The dCas13–ALKBH5-MUT cannot affect the 15q-TERRA m^6^A modification or 15q-TERRA levels, and the 6q-TERRA m^6^A modification or expression levels were not changed with all of these treatments (Figure 2J, K). Altogether, these results reveal that the first RRACH motif of 15q-TERRA is the key site to be m^6^A modified by METTL3 and regulates 15q-TERRA stability.

### YTHDC1 protects m^6^A-modified TERRA

**Figure 3.**
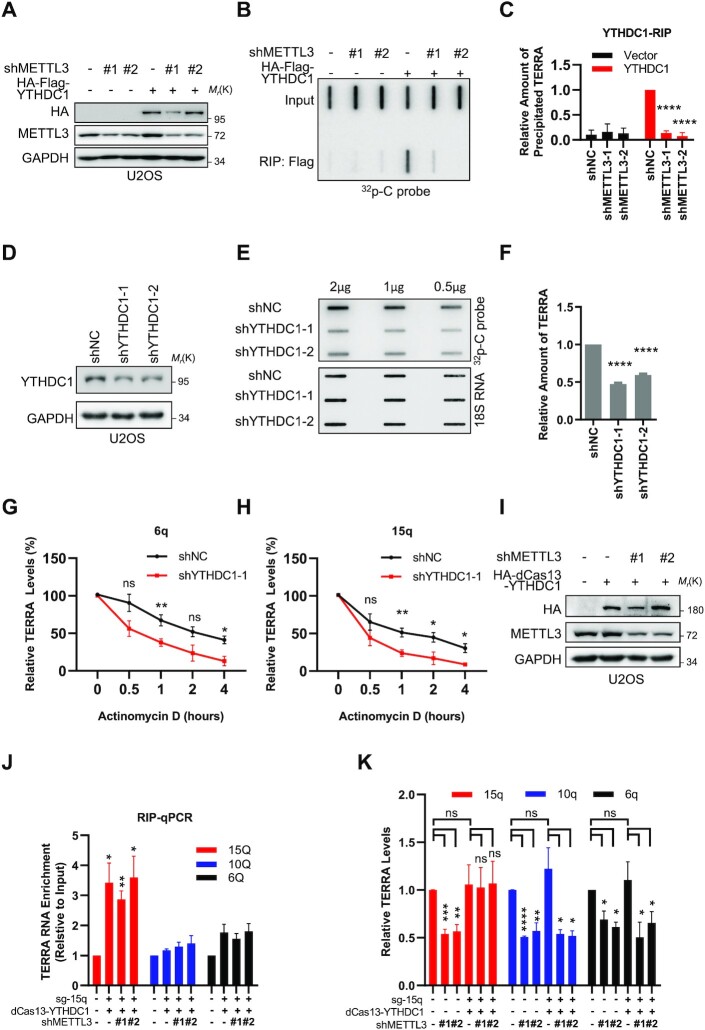
YTHDC1 protects m^6^A-modified TERRA. (**A**) Western blot analysis of the HA-Flag-YTHDC1 overexpression level in the indicated U2OS cells. (**B**) YTHDC1 binds to m^6^A-modified TERRA. RIP was performed using anti-Flag beads in METTL3-depleted and control U2OS cells expressing HA-Flag-YTHDC1. TERRA in precipitates was detected by ^32^P-C probe. The same amount of TERRA was loaded as indicated by ‘input’. (**C**) Quantification of (B). The relative amount of TERRA was calculated as elution/input, and normalized to the shNC group with YTHDC1 overexpressed. (**D**) Western blot analysis of YTHDC1 knockdown efficiency in U2OS cells. (**E**) TERRA decreases in YTHDC1-depleted U2OS cells. Slot-blot was performed to determine the TERRA level, with 18S RNA as loading control. (**F**) Quantification of (E). The amount of TERRA was calculated as TERRA intensity/18S RNA intensity, and then normalized to the shNC group. (**G, H**) TERRA degrades rapidly in YTHDC1-depleted U2OS cells. Transcription was inhibited by 5 μg/ml AcTD in YTHDC1-depleted and control U2OS cells. The TERRA levels of the 6q chromosome (G) and 15q chromosome (H) were determined by RT–qPCR. The 18S RNAs were used for normalization. (**I**) Western blot analysis of HA-dCas13–YTHDC overexpression in the indicated U2OS cells. (**J**) dCas13–YTHDC1 binds to 15q-TERRA independent of m^6^A modification. dCas13–YTHDC1 was artificially guided to 15q-TERRA by sg-15q in METTL3-depleted and control U2OS cells. RIP was performed by pulling down dCas13–YTHDC1. TERRA was detected by RT–qPCR. (**K**) dCas13–YTHDC1 rescues the 15q-TERRA level in METTL3-depleted U2OS cells. Cells were treated as in (J), and relative 15q-TERRA, 10q-TERRA or 6q-TERRA levels were determined by RT–qPCR without RIP. The 18S RNAs were used for normalization. (*P <0.05, **P <0.01, ***P <0.001, ****P <0.0001).

We next investigated the mechanism by which m^6^A modification maintains the stability of TERRA. We noticed that m^6^A modification of RNA is mainly recognized by the YTH domain family (YTHDF1, 2 and 3) and YTH domain-containing proteins (YTHDC1 and 2). Since YTHDFs and YTHDC2 are primarily located in the cytoplasm while YTHDC1 is in the nucleus as is TERRA (35), YTHDC1 is more likely to be involved in protecting m^6^A-modified TERRA. By YTHDC1 RIP assay, we observed that YTHDC1 binds to TERRA, and the precipitated TERRA decreased after METTL3 knockdown (Figure 3A–C). In addition, we observed that TERRA is decreased significantly in YTHDC1-deficient cells (Figure 3D–F), and the 6q- and 15q-TERRA half-lives are shortened in YTHDC1-deficient cells (Figure 3G, H). These results demonstrate that YTHDC1 reduction leads to faster TERRA degradation. To further prove that the binding of YTHDC1 stabilizes TERRA, we artificially guided YTHDC1 to 15q-TERRA directly using the dCas13 system with 15q-TERRA sgRNAs (sg-15q). Through RIP assay, we found that dCas13–YTHDC1 binds to 15q-TERRA but not to others (such as 10q-TERRA and 6q-TERRA) independently of m^6^A modification (Figure 3I, J). Consistently, only the 15q-TERRA expression level in shMETTL3 cells is maintained as high as in the control in the presence of the dCas13–YTHDC1 system but not 10q- or 6q-TERRA, which decreased in shMETTL3 cells the same as in cells without the dCas13–YTHDC1 system (Figure 3K). Altogether, these data support the hypothesis that YTHDC1 binds to and stabilizes m^6^A-modified TERRA.

### METTL3 deficiency interrupts telomeric homologous recombination in ALT cells

**Figure 4.**
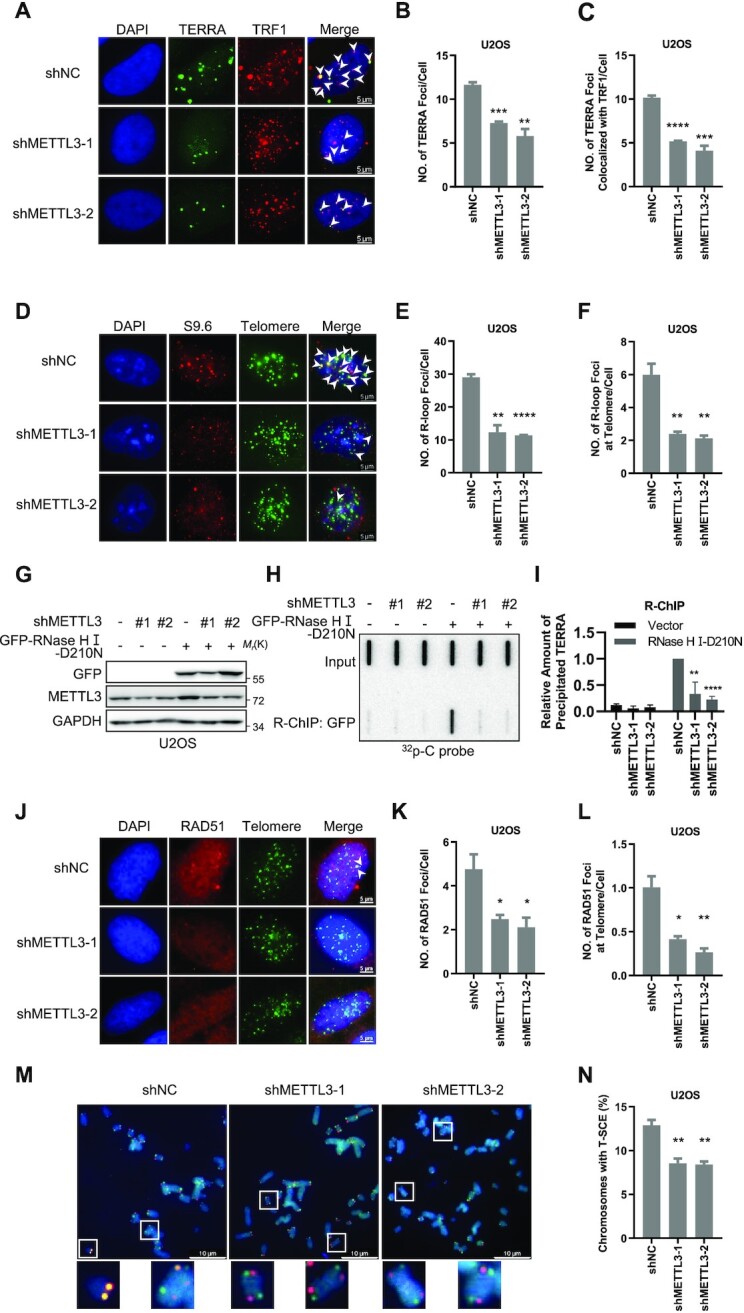
METTL3 deficiency interrupts telomeric HR in ALT cells. (**A**) TERRA foci at telomeres decrease in METTL3-depleted U2OS cells. TERRA and TRF1 were detected by C probe and anti-TRF1 antibody. respectively. Scale bars, 5 μm. (**B**) Quantification of (A). The total numbers of TERRA foci per cell were counted (*n* ≥100 cells × 3 repeats). (**C**) Quantification of (A). The numbers of TERRA foci co-localized with TRF1 were counted (*n* ≥100 cells × 3 repeats). (**D**) R-loop foci at telomeres decrease in METTL3-depleted U2OS cells. R-loops and telomeres were detected by anti-S9.6 antibody and C probe, respectively. Scale bars, 5 μm. (**E**) Quantification of (D). The mean numbers of R-loop foci per cell were counted (*n* ≥100 cells × 3 repeats). (**F**) Quantification of (D). The mean numbers of R-loop foci co-localized with telomeres were counted (*n* ≥100 cells × 3 repeats). (**G**) Western blot analysis of GFP–RNase H-mut overexpression in the indicated U2OS cells. (**H**) GFP–RNase HI-D210N binds to TERRA R-loops. R-ChIP was performed using anti-GFP beads in METTL3-depleted and control U2OS cells expressing GFP–RNase HI-D210N. TERRA in precipitates was detected by ^32^P-C probe. The same amount of TERRA was loaded as indicated by ‘input’. (**I**) Quantification of (H). The amount of TERRA was calculated as the TERRA intensity of elution/TERRA intensity of input, and then normalized to shNC with the RNase HI-D210N overexpression group. (**J**) RAD51 foci at telomeres decrease in METTL3-depleted U2OS cells. RAD51 and telomeres were detected by anti-RAD51 antibody and C probe, respectively. Scale bars, 5 μm. (**K**) Quantification of (J). The mean numbers of RAD51 foci per cell were counted (*n* ≥100 cells × 3 repeats). (**L**) Quantification of (J). The mean numbers of RAD51 foci co-localized with telomeres were counted (*n* ≥100 cells × 3 repeats). (**M**) T-SCE frequency decreases in METTL3-depleted U2OS cells. Telomeric sister chromatin exchange was detected by CO-FISH in the indicated U2OS cells. The C-rich and G-rich strands of telomeres are visualized with Cy3-labeled G-rich probe (red) or FITC-labeled C-rich probe (green), respectively. Yellow dots indicate the presence of T-SCE. Scale bars, 10 μm. (**N**) Quantification of (M). The percentages of chromosomes with T-SCE were calculated (*n* ≥670 chromosomes). (*P <0.05, **P <0.01, ***P <0.001, ****P <0.0001).

It is well known that TERRA is a type of telomere-associated long non-coding RNA, which forms an R-loop and plays a critical role in telomere maintenance (16,20). We studied whether m^6^A-modified TERRA is involved in R-loop formation. First, IF-RNA FISH results showed that both total TERRA foci and TERRA foci at telomeres are decreased in METTL3-deficient cells (Figure 4A–C; Supplementary Figure S2A, B). Next, IF-FISH with S9.6 antibody, which specifically recognizes RNA–DNA hybrids (Supplementary Figure S2C, D), revealed that METTL3 depletion leads to a significant decrease of S9.6 foci universally in cells and at telomeres in both U2OS and VA13 cells (Figure 4D–F; Supplementary Figure S2E–H). Furthermore, we overexpressed a catalytically active mutant of RNase H (D210N), which recognizes and binds to the R-loop but does not degrade it, in U2OS cells followed by RNase H ChIP (R-ChIP) (49,50). We efficiently captured R-loops containing TERRA, and the amount of precipitated TERRA decreased with METTL3 deficiency (Figure 4G–I). Given that YTHDC1 binds to m^6^A-modified TERRA (Figure 3B, C), it is also expected to be associated with telomeres as m^6^A-modified TERRA engages in R-loops. The YTHDC1 ChIP experiment showed that it does bind to telomeres, which is also regulated by the METTL3 expression level (Supplementary Figure S2I, J). These results suggest that m^6^A-modified TERRA forms R-loops with telomeres.

A previous study showed that the m^6^A-modulated RNA–DNA hybrids are involved in DNA damage response and DSB repair by recruiting RAD51 for HR (40). This raises the possibility that m^6^A-modified TERRA regulates telomeric HR through forming R-loops with telomeres. RPA1 and Rad51 are single-stranded DNA (ssDNA) binding proteins which play critical roles in HR. RPA1 rapidly binds to and protects ssDNA at DSB sites (51–53). Subsequently, RAD51 replaces RPA1 at ssDNA and promotes its invasion into homologous dsDNA (54–56). We found that METTL3 depletion increases RPA1 foci, whereas it decreases Rad51 foci both universally and at telomeres in U2OS and VA13 cells (Figure 4J–L; Supplementary Figure S3A–I). It seems that METTL3 deficiency impairs the switch from RPA1 to RAD51 both universally and at telomeres. It suggests that METTL3 deletion inhibits strand invasion of telomeric DNA in ALT cells. As a result, telomeric HR would be suppressed. Since HR at telomeres leads to T-SCE (57), we performed CO-FISH to detect the frequency of T-SCEs. Indeed, the results showed that the frequency of T-SCEs is decreased in METTL3-deficient cells (Figure 4M, N). These observations demonstrate that METTL3 deficiency reduces telomeric R-loops and impairs RAD51 co-localization with telomeres, subsequently inhibiting strand invasion and interrupting telomeric HR in ALT cells. In the same way, IF-FISH results showed that YTHDC1 depletion significantly reduces the telomeric R-loops and the recombinase RAD51 foci at telomeres, which is similar to METTL3 deficiency (Supplementary Figure S3J–O). In summary, these observations suggest that the m^6^A-modified TERRA forms R-loops with telomeres, and the absence of METTL3 impairs telomeric HR.

### METTL3 depletion triggers telomere dysfunction

**Figure 5.**
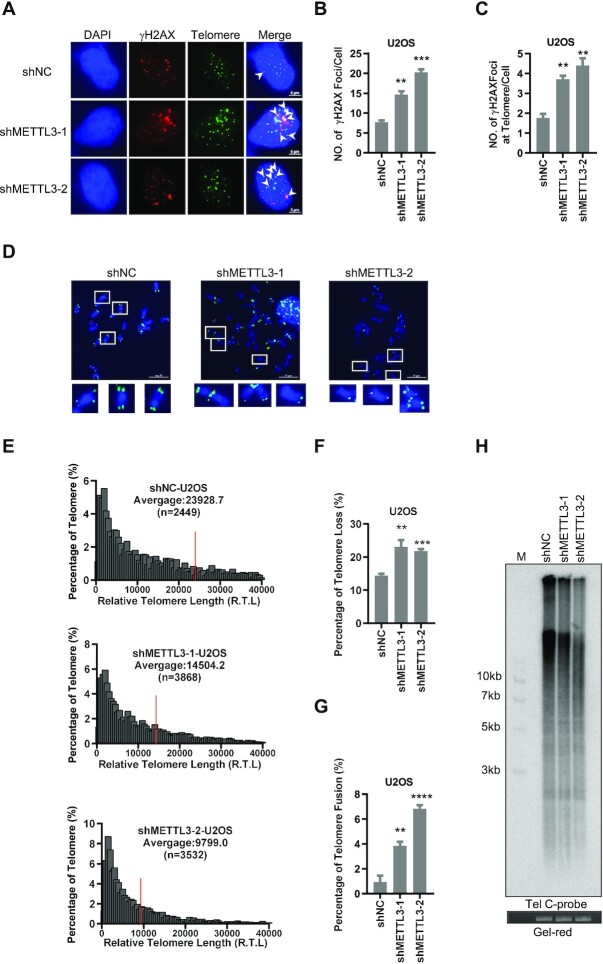
METTL3 depletion triggers telomere dysfunction. (**A**) γH2AX foci increase in METTL3-depleted U2OS cells. γH2AX and telomeres were detected by anti-γH2AX antibody and C probe, respectively. Scale bars, 5 μm. (**B**) Quantification of (A). The mean numbers of γH2AX foci per cell were counted (*n* ≥100 cells × 3 repeats). (**C**) Quantification of (A). The mean numbers of γH2AX foci co-localized with telomeres were counted (*n* ≥100 cells × 3 repeats). (**D**) q-FISH detection of telomeres on metaphase spreads of METTL3-depleted U2OS cells. Scale bars, 10 μm. (**E**) Quantification of (D). The relative telomere lengths were quantified as the fluorescent intensity. The numbers of quantified telomeres are indicated by ‘*n*’. Red lines indicate the average length of telomeres. (**F**) Quantification of (D). The percentages of chromosomes with one or more telomere free ends were calculated (*n* ≥1000 chromosomes). (**G**) Quantification of (D). The percentages of telomere end-to-end fusion were calculated (*n* ≥1000 chromosomes). (**H**) Telomere length shortened in METTL3-depleted U2OS cells. Telomere restriction fragment assay was performed using ^32^P-C probe. The gel red staining was used as loading control. (**P <0.01, ***P <0.001, ****P <0.0001).

Telomeric HR is one of the principal mechanisms for maintenance of telomere length and stability (58–62). We thus investigated whether METTL3 deficiency affects the end protection function of telomeres. The results showed that there is a significant increase in γH2AX foci co-localizing with telomeres after METTL3 depletion in U2OS and VA13 cells, implying increased TIFs (Figure 5A–C; Supplementary Figure S4A–C). q-FISH was performed in METTL3-depleted U2OS and VA13 cells to determine the telomere length and stability. The results showed that the telomere length is shortened upon METTL3 deficiency (Figure 5D, E; Supplementary Figure S4D–F). Consistently, the TRF displayed a similar result (Figure 5H). Meanwhile, telomere loss and chromosome end-to-end fusion are increased (Figure 5D, F, G; Supplementary Figure S4E, G, H). These results suggest that METTL3 deficiency leads to telomere dysfunction.

Moreover, YTHDC1 deletion also increases the level of γH2AX at telomeres (Supplementary Figure S4I–K), suggesting that YTHDC1 deletion is similar to METTL3 deficiency which leads to telomere instability. Given that mRNAs are widely modified and regulated by m^6^A modification, it is possible that METTL3 affects telomere stability by modulating the metabolism of shelterin mRNA, such as TRF1, TRF2 and POT1. Hence, we performed western blot to detect the protein expression levels after METTL3 depletion. We found that knockdown of METTL3 does not affect the expression levels of shelterin proteins (Supplementary Figure S4L), eliminating the possibility that telomere dysfunction caused by METTL3 is due to TRF1, TRF2 or POT1 deficiency.

### Restoring R-loop levels rescues interrupted HR

**Figure 6.**
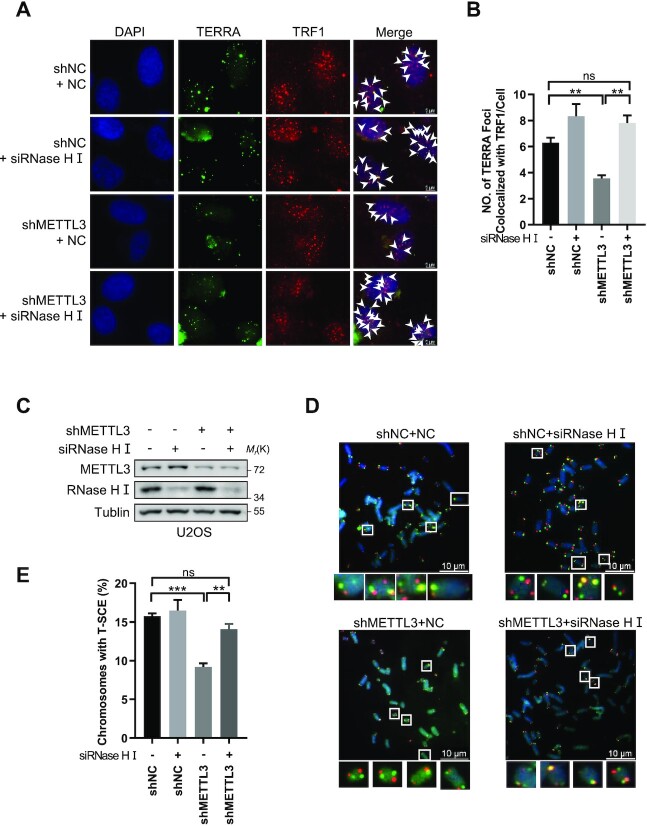
RNase HI knockdown rescues interrupted HR. (**A**) TERRA foci at telomeres are rescued by RNase HI knockdown. RNase HI was knocked down by siRNAs in METTL3-depleted and control U2OS cells as indicated. TERRA and TRF1 were detected by C probe and anti-TRF1 antibody, respectively. Scale bars, 5 μm. (**B**) Quantification of (A). The numbers of TERRA foci co-localized with telomeres were counted (*n* ≥100 cells × 3 repeats). (**C**) Western blot analysis of RNase HI knockdown efficiency in the indicated U2OS cells. (**D**) T-SCE frequency is rescued by RNase HI knockdown. RNase HI was knocked down by siRNAs in METTL3-depleted and control U2OS cells as indicated. Telomeres were detected by CO-FISH assay. The C-rich and G-rich strands of telomeres are visualized with Cy3-labeled G-rich probe (red) or FITC-labeled C-rich probe (green), respectively. Yellow dots indicate the presence of T-SCE. Scale bars, 10 μm. (**E**) Quantification of (D). The percentages of chromosomes with T-SCE were calculated (*n* ≥1000 chromosomes). All values are means ± SEM of three independent experiments. (*P <0.05, **P <0.01, ***P <0.001).

If the HR disruption and telomere dysfunction induced by METTL3 deficiency are due to lack of telomeric R-loops, it is speculated that restoring R-loops by deletion of RNase HI in METTL3-deficient cells would rescue the phenomenon. To test this hypothesis, we knocked down RNase HI in METTL3-deficient U2OS cells and observed that TERRA foci at telomeres in METTL3-deficient cells are rescued to the normal level (Figure 6A–C). Similarly, we observed that deletion of RNase HI also rescued telomeric T-SCE frequency, which is reduced by METTL3 deficiency, to the normal level in U2OS cells (Figure 6D, E). Hence, METTL3 deficient-induced telomere HR disruption, which is a sign of telomere instability, can be restored by rescuing telomeric R-loops through RNase HI deletion.

Our results above reveal that METTL3 is very important for telomere stability in ALT cells, so we wondered whether loss of METTL3 affects the growth of ALT tumor cells. Hence, cell viability of ALT cancer cells (U2OS) and telomerase-positive cells [CAL27, which are established from poorly differentiated squamous cell carcinoma of the tongue, is a telomerase-positive cell line (63)] were detected for 6 days after METTL3 deletion. The results showed that the viability of U2OS but not CAL27 cells decreases significantly after METTL3 deletion (Supplementary Figure S5A–C). In addition, colony formation assay showed that fewer colonies form in METTL3-deficient U2OS cells than in control cells (Supplementary Figure S5D, E). These data indicate that lack of METTL3 leads to growth inhibition in ALT cancer cells.

## DISCUSSION

In this study, we demonstrated that the methyltransferase METTL3 could install the m^6^A modification at TERRA subtelomeric regions, which is recognized and protected by YTHDC1 later on. The m^6^A-modified TERRA forms R-loops with telomeres, and the absence of METTL3 impairs telomeric HR, telomere shortening and instability. These findings highlight the importance of RNA epigenetics in maintaining telomere integrity.

### METTL3-mediated m^6^A modification regulates the stability of TERRA

TERRA is transcribed from multiple chromosome ends and is comprised of subtelomeric regions and telomeric repeats. A large number of studies have focused on the function of TERRA telomeric repeats but little is known about its subtelomeric regions. Our study proved that m^6^A modification mediated by METTL3 localizes to TERRA subtelomeric regions and regulates the stability of TERRA by the following observations. First, m^6^A signal is detectable on TERRA, and TERRA can be pulled down by m^6^A antibody, both of which diminished after METTL3 knockdown, suggesting that METTL3 catalyzes m^6^A modification on TERRA (Figure 1). Secondly, the RNase digestion assay demonstrates that m^6^A modification mainly localizes at TERRA subtelomeric regions (Figure 1). Thirdly, reduced m^6^A modification by either METTL3 depletion or targeted dCas13–ALKBH5 promotes endogenous TERRA degradation, and m^6^A modification stabilizes exogenous TERRA *in vitro* (Figure 2). Thus, we proposed that m^6^A modification at subtelomeric regions might be essential for the stability of TERRA.

It is widely accepted that RNA-binding proteins could protect RNAs from undesired digestion by RNase (64). Recently, we and other groups have discovered that YTHDC1 can protect m^6^A-RNAs from degradation by associating with them (40,65). Consistent with this observation, our results showed that depletion of YTHDC1 decreases the half-lives of TERRA transcripts, and artificially tethering YTHDC1 to 15q-TERRA by the dCas13 system can rescue the METTL3 deficiency-induced 15q-TERRA decrease (Figure 3). These findings allow us to propose that YTHDC1 protects m^6^A-modified TERRA from being degraded.

### Dual roles of TERRA during telomere stability

A myriad of studies indicate that TERRA plays a physiologically relevant role in the overall maintenance of telomeres and/or telomere function (47,66,67). It is reported that TERRA can hybridize with telomere repeats to form R-loops, which promote telomeric HR and synthesis of new telomeric DNA. Here, we found that the decreased TERRA levels upon knockdown of METTL3 will increase the TIFs, telomere loss and telomere fusion, and therefore shorten the telomere length (Figure 5). In contrast, it has also been proposed that abnormal elevation of TERRA levels may induce telomere aberrations such as telomere-free ends and telomere shortening by interfering with telomere replication (18,20,68,69). The seemingly opposite dual roles of TERRA in telomere stability could be interpreted as only a moderate number of TERRAs are required for telomere homeostasis whereas excess TERRA has the opposite effect by forming redundant R-loops, which impede the progression of the telomere replication fork and induce DNA damage at telomeres. In this scenario, METTL3 and m^6^A play important roles to maintain optimal TERRA levels resulting in a favorable number of R-loops at telomeres, which support telomeric HR without endangering telomere integrity.

### New function of METTL3 and m^6^A in ALT telomere maintenance

Our study also supports previous finding that METTL3 and m^6^A are vital to maintain genomic stability. It has been reported that m^6^A-modified RNAs are required for recruitment of Pol κ to sites of DNA damage to participate in nucleotide excision repair (NER) (70). In addition, phosphorylated METTL3 methylates the RNAs associated with damaged DNA, which is protected by YTHDC1 and forms RNA–DNA hybrids to promote HR-mediated repair (40). We demonstrated that METTL3 depletion-induced telomere dysfunction is mainly caused by the decrease of HR due to lack of TERRA. In the same way as DSB sites need DNA damage-associated RNAs, telomeres need the TERRA to form R-loops and facilitate HR. We also found that YTHDC1 is necessary for TERRA protection similar to the RNAs at DSB sites. METTL3 depletion-induced telomere instability can be rescued by knockdown of RNase HI (Figure 6). In addition, we found that depletion of METTL3 does not affect the amounts of TRF1, TRF2 and POT1. These results excluded the possibility that METTL3 and m^6^A affect telomere stability through altering the metabolism of shelterin transcripts.

As compared with telomerase-positive cancer cells, ALT cancer cells have a large amount of intrinsic DNA damage and maintain telomere length depending on frequent HR (11,60). Thus, inhibition of DNA damage repair pathways is an effective therapy for ALT cancer, for example an ATR targeting inhibitor has been shown to block ALT cell proliferation (71–73). In this study, we found that deletion of METTL3 also inhibits telomeric HR by reducing R-loop formation and RAD51 recruitment to telomeres (Figure 4), leading to progressive telomere shortening and dysfunction (Figure 5). Therefore, our results indicate that inhibition or deletion of METTL3 is potentially a new route fo ALT cancer therapy. It is worth searching for feasible ways to inhibit or delete METTL3 clinically.

## DATA AVAILABILITY

All data have been included in the manuscript, figures and supplemental data.

## Supplementary Material

gkac1027_Supplemental_File
