# Supplementary material for: METTL3-mediated m6A modification stabilizes TERRA and maintains telomere stability
Source: Nucleic Acids Res. 2022 Nov 18;50(20):11619–34. doi: 10.1093/nar/gkac1027 (PMC9723618; doi:10.1093/nar/gkac1027)

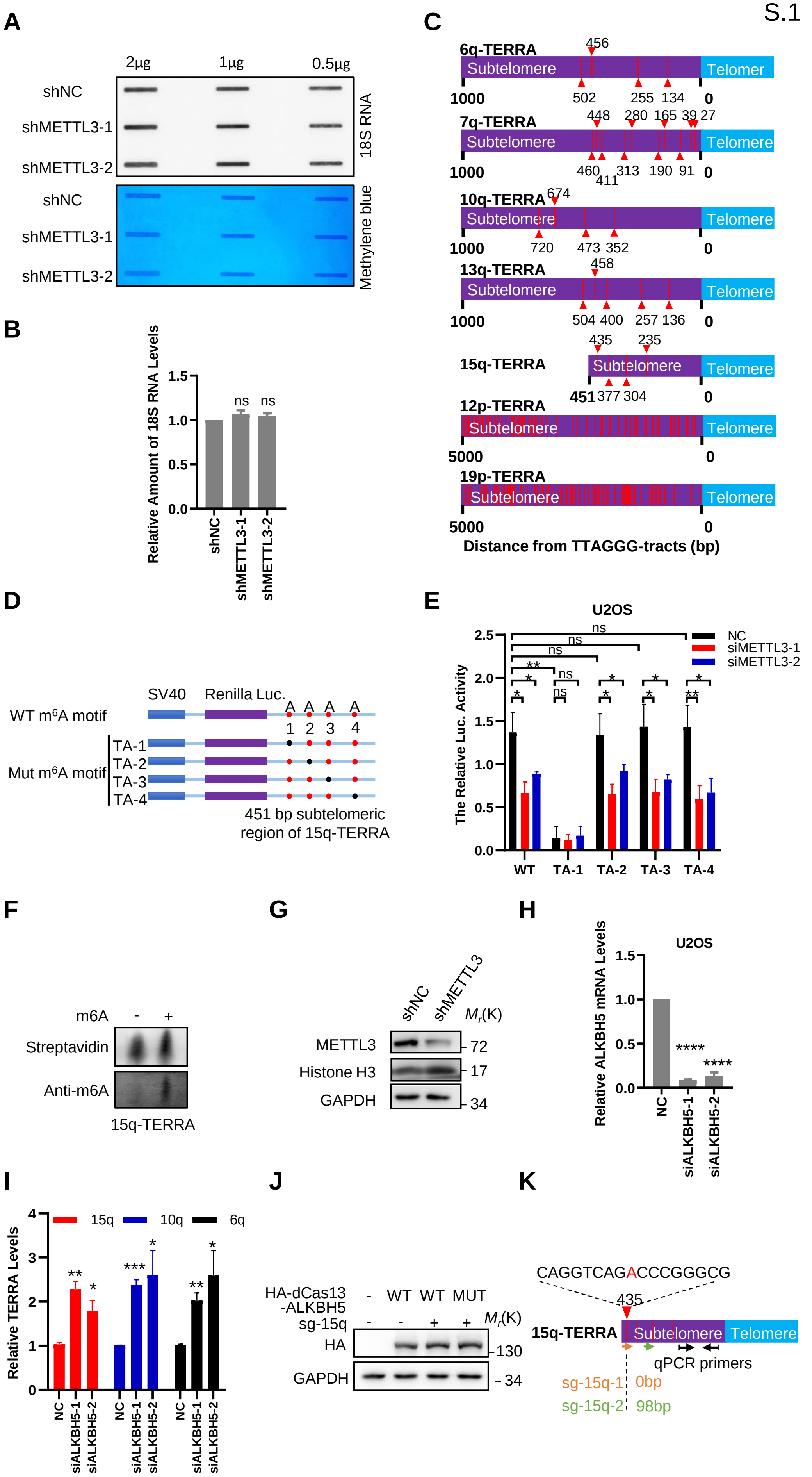
A
2μg 1μg 0.5μg
shNC
shMETTL3-1
shMETTL3-2
18S RNA
Methylene blue
B
Relative Amount of 18S RNA Levels
ns ns
shNC shMETTL3-1 shMETTL3-2
C
6q-TERRA
7q-TERRA
10q-TERRA
13q-TERRA
15q-TERRA
12p-TERRA
19p-TERRA
Subtelomere
Telomere
Distance from TTAGGG-tracts (bp)
D
SV40
Renilla Luc.
WT m6A motif
Mut m6A motif
TA-1 TA-2 TA-3 TA-4
451 bp subtelomeric region of 15q-TERRA
E
U2OS
The Relative Luc. Activity
NC siMETTL3-1 siMETTL3-2
WT TA-1 TA-2 TA-3 TA-4
F
m6A
Streptavidin
Anti-m6A
15q-TERRA
G
shNC shMETTL3
Mr(K)
METTL3
Histone H3
GAPDH
H
U2OS
Relative ALKBH5 mRNA Levels
NC siALKBH5-1 siALKBH5-2
I
Relative TERRA Levels
15q 10q 6q
J
HA-dCas13-ALKBH5
sg-15q
HA
GAPDH
K
CAGGTCAGACCCGGGCG
15q-TERRA
qPCR primers
sg-15q-1 0bp
sg-15q-2 98bp

Supplementary Figure S1. TERRA m6A modification sites information. **(A)** The levels of 18S RNA do not change upon METTL3 depletion. Slot blot was performed to determine 18S RNA levels, with Methylene blue staining as loading control. **(B)** Quantification of (A). The relative intensity of 18S RNA signals. **(C)** Schematic illustration of RRACH motifs at subtelomeres. Each red line represents a RRACH motif. **(D)** Schematic diagram of 15q-TERRA mutations on Renilla luciferase reporter plasmids. Wild type or m6A consensus sequence mutants (A to T) of 451 bp subtelomere 15q-TERRA were cloned to Renilla luciferase reporter, resulting in WT motif, TA-1, TA-2, TA-3 and TA-4. **(E)** TA-1 mutation reduces Renilla luciferase activity irrespective of METTL3. U2OS cells, which have been depleted METTL3 by siRNA, were transfected with luciferase reporter plasmids in (D). Relative luciferase activity was measured and normalized to firefly luciferase activity. **(F)** Biotin labeled 51 nt of 15q-TERRA fragments containing wild type TA-1 site were synthesized with or without m6A modification. The RNAs were separated by urea-PAGE gel and staining with streptavidin and m6A antibody. **(G)** Western Blot analysis of METTL3 knockdown efficiency and nuclear marker (Histone H3) in U2OS cells lysate. **(H)** The relative ALKBH5 mRNA levels in ALKBH5 depleted and control U2OS cells. **(I)** The TERRA on 15q, 10q and 6q chromosomes in ALKBH5 depleted U2OS cells were detected by RT-qPCR. The 18S RNAs were used for normalization. **(J)** Western Blot analysis of HA-dCas13-ALKBH5 overexpression levels in indicated U2OS cells. The sgRNAs of 15q-TERRA (sg-15q) include sg-15q-1 and sg-15q-2. **(K)** Schematic illustration of sg-15q targeting sites. sg-15q-1 targets at the first m6A site whereas sg-15q-2 targeting site is 98 bp away from the first m6A site. (* $P<0.05$, ** $P<0.01$, *** $P<0.001$, ****$P<0.0001$).

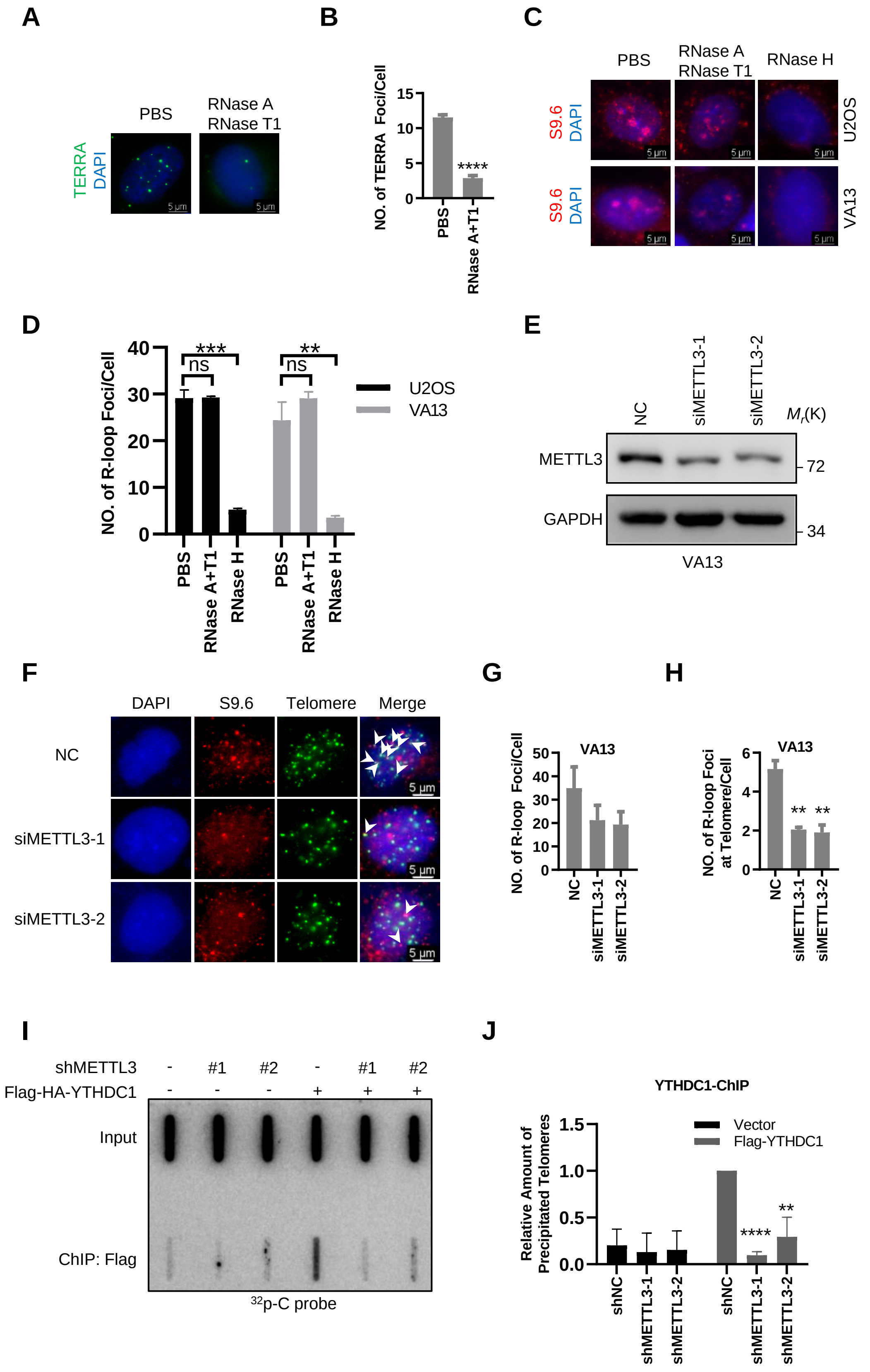
A
PBS
RNase A
RNase T1
TERRA
DAPI
5 μm
B
NO. of TERRA Foci/Cell
15
10
5
0
****
PBS
RNase A+T1
C
PBS
RNase A
RNase T1
RNase H
S9.6
DAPI
U2OS
VA13
5 μm
D
NO. of R-loop Foci/Cell
40
30
20
10
0
***
ns
**
ns
U2OS
VA13
PBS
RNase A+T1
RNase H
E
NC
siMETTL3-1
siMETTL3-2
M_r(K)
METTL3
72
GAPDH
34
VA13
F
DAPI
S9.6
Telomere
Merge
NC
siMETTL3-1
siMETTL3-2
5 μm
G
VA13
NO. of R-loop Foci/Cell
50
40
30
20
10
0
NC
siMETTL3-1
siMETTL3-2
H
VA13
NO. of R-loop Foci at Telomere/Cell
6
4
2
0
**
**
NC
siMETTL3-1
siMETTL3-2
I
shMETTL3
-
#1
#2
-
#1
#2
Flag-HA-YTHDC1
-
-
-
+
+
+
Input
ChIP: Flag
32p-C probe
J
YTHDC1-ChIP
Relative Amount of Precipitated Telomeres
1.5
1.0
0.5
0.0
Vector
Flag-YTHDC1
****
**
shNC
shMETTL3-1
shMETTL3-2
shNC
shMETTL3-1
shMETTL3-2

Supplementary Figure S2. m6A-TERRA forms R-loops with telomeres in ALT cells. **(A)** RNA FISH detection of TERRA. U2OS cells were treated with or without RNase A and RNase T1 (DNase free) before fixed. RNA FISH was performed using telomere C probe, Scale bars, 5 μm. **(B)** Quantification of (A). The mean numbers of TERRA foci per cell were counted (n≥100 cells × 3 repeats). **(C)** S9.6 antibody binds to R-loops specifically. Cells were treated with RNase H to remove R-loops (negative control), or with RNase A and RNase T1 to remove free RNAs. Then, R-loops were detected by anti-S9.6 antibody in U2OS cells and VA13 cells. Scale bars, 5 μm. **(D)** Quantification of (C). The mean numbers of R-loop foci per cell were counted (n≥100 cells × 3 repeats). **(E)** Western blot analysis of METTL3 knockdown efficiency in VA13 cells transfected with siRNAs. **(F)** R-loop foci at telomeres decrease in METTL3 depleted VA13 cells. R-loops and telomeres were detected by anti-S9.6 antibody and C probe respectively. Scale bars, 5 μm. **(G)** Quantification of (F). The mean numbers of R-loop foci per cell were counted (n≥100 cells×3 repeats). **(H)** Quantification of (F). The mean numbers of R-loop foci colocalized with telomeres were counted (n≥100 cells×3 repeats). **(I)** YTHDC1 binds to telomere R-loop. HA-Flag-YTHDC1 was transfected into U2OS cells and ChIP was performed using anti-Flag beads in METTL3 depleted and control U2OS cells. Telomeres in precipitates were detected by ^{32}p-C probe. Same amount of telomere was loaded as indicated by "input". **(J)** Quantification of (I). The amount of TERRA was calculated as telomere-intensity of elution/telomere-intensity of input, and then normalized to shNC with YTHDC1 overexpression group. (** P<0.01, *** P<0.001, ****P<0.0001).

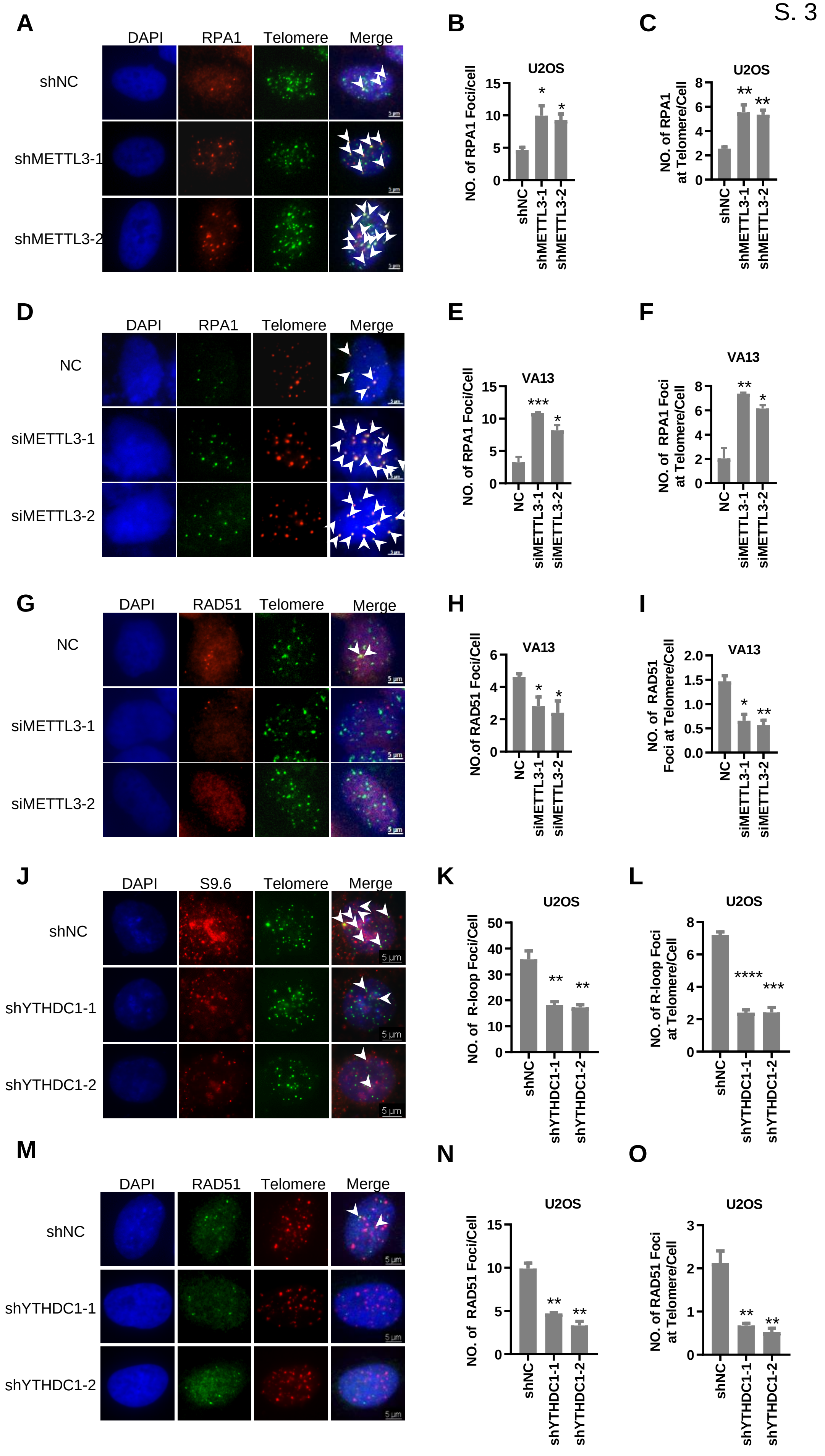
A
DAPI
RPA1
Telomere
Merge
shNC
shMETTL3-1
shMETTL3-2
B
U2OS
NO. of RPA1 Foci/cell
C
U2OS
NO. of RPA1 at Telomere/Cell
D
NC
siMETTL3-1
siMETTL3-2
E
VA13
NO. of RPA1 Foci/Cell
F
NO. of RPA1 Foci at Telomere/Cell
G
RAD51
H
NO.of RAD51 Foci/Cell
I
NO. of RAD51 Foci at Telomere/Cell
J
S9.6
shYTHDC1-1
shYTHDC1-2
5 μm
K
NO. of R-loop Foci/Cell
L
NO. of R-loop Foci at Telomere/Cell
M
N
NO. of RAD51 Foci/Cell
O
NO. of RAD51 Foci at Telomere/Cell

Supplementary Figure S3. METTL3 or YTHDC1 deficiency interrupts telomeric HR in ALT cells. **(A)** RPA1 foci at telomeres increase in METTL3 depleted U2OS cells. RPA1 and telomeres were detected by anti-RPA1 antibody and C probe respectively. Scale bars, 5 μm. **(B)** Quantification of (A). The mean numbers of RPA1 foci per cell were counted. **(C)** Quantification of (A). The mean numbers of RPA1 foci colocalized with telomeres were counted. **(D-F)** The same experiment and quantification as (A-C) was repeated in the VA13 cells. **(G)** RAD51 foci at telomeres decrease in METTL3 depleted VA13 cells. RAD51 and telomere were detected by anti-RAD51 antibody and C probe respectively. Scale bars, 5 μm. **(H)** Quantification of (G). The mean numbers of RAD51 foci per cell were counted. **(I)** Quantification of (G). The mean numbers of RAD51 foci colocalized with telomeres were counted. **(J)** R-loop foci at telomeres decrease in YTHDC1 depleted U2OS cells. R-loops and telomeres were detected by anti-S9.6 antibody and C probe respectively. Scale bars, 5 μm. **(K)** Quantification of (J). The mean numbers of R-loop foci per cell were counted. **(L)** Quantification of (J). The mean numbers of R-loop foci colocalized with telomeres were counted. **(M)** RAD51 foci at telomeres decrease in YTHDC1 depleted U2OS cells. RAD51 and telomeres were detected by anti-RAD51 antibody and C probe respectively. Scale bars, 5 μm. **(N)** Quantification of (M). The mean numbers of RAD51 foci per cell were counted. **(O)** Quantification of (M). The mean numbers of RAD51 foci colocalized with telomeres were counted. (For all panels, $n \geq 100$ cells × 3 repeats, * $P<0.05$, ** $P<0.01$, *** $P<0.001$, ****$P<0.0001$).

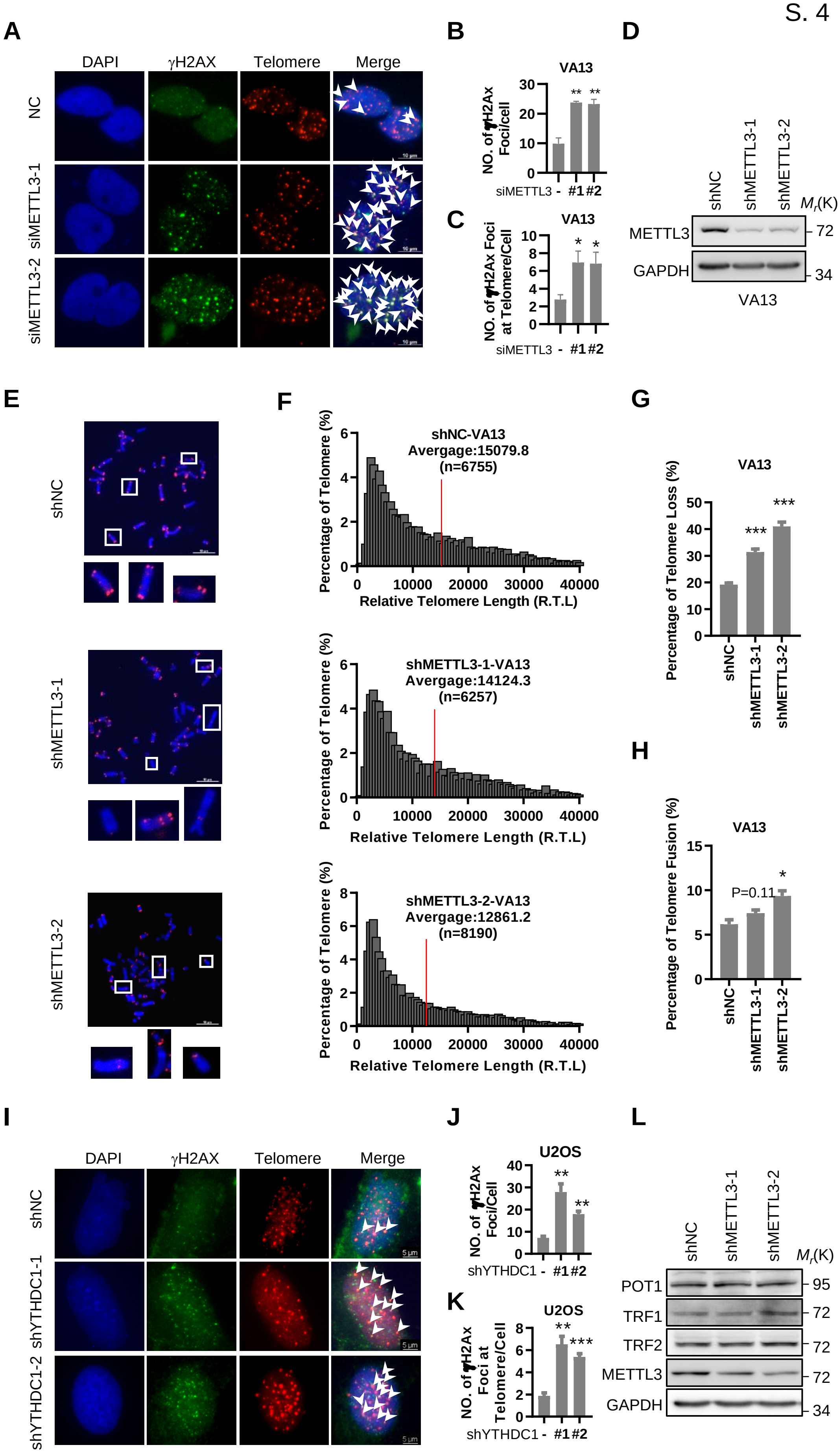
A
DAPI
γH2AX
Telomere
Merge
NC
siMETTL3-1
siMETTL3-2
B
VA13
NO. of γH2Ax Foci/cell
siMETTL3 - #1 #2
C
VA13
NO. of γH2Ax Foci at Telomere/Cell
siMETTL3 - #1 #2
D
shNC
shMETTL3-1
shMETTL3-2
M_r(K)
METTL3
GAPDH
72
34
VA13
E
shNC
shMETTL3-1
shMETTL3-2
F
shNC-VA13
Avergage:15079.8
(n=6755)
shMETTL3-1-VA13
Avergage:14124.3
(n=6257)
shMETTL3-2-VA13
Avergage:12861.2
(n=8190)
Percentage of Telomere (%)
Relative Telomere Length (R.T.L)
G
VA13
Percentage of Telomere Loss (%)
shNC
shMETTL3-1
shMETTL3-2
H
VA13
P=0.11
Percentage of Telomere Fusion (%)
shNC
shMETTL3-1
shMETTL3-2
I
DAPI
γH2AX
Telomere
Merge
shNC
shYTHDC1-1
shYTHDC1-2
J
U2OS
NO. of γH2Ax Foci/Cell
shYTHDC1 - #1 #2
K
U2OS
NO. of γH2Ax Foci at Telomere/Cell
shYTHDC1 - #1 #2
L
shNC
shMETTL3-1
shMETTL3-2
M_r(K)
POT1
TRF1
TRF2
METTL3
GAPDH
95
72
72
72
34

Supplementary Figure S4. METTL3 depletion triggers telomere dysfunction. **(A)** γH2AX foci increase in METTL3 depleted VA13 cells. γH2AX and telomeres were detected by anti-γH2AX antibody and C probe respectively. Scale bars, 10 μm. **(B)** Quantification of (A). The mean numbers of γH2AX foci per cell were counted (n≥100 cells × 3 repeats). **(C)** Quantification of (A). The mean numbers of γH2AX foci colocalized with telomeres were counte (n≥100 cells × 3 repeats). **(D)** Western blot analysis of METTL3 knockdown efficiency in VA13 cells transfected with shRNAs. **(E)** Q-FISH of telomeres on metaphase spreads of METTL3 depleted VA13 cells. Scale bars, 10 μm. **(F)** Quantification of (E). The relative telomere lengths were quantified as the fluorescent intensity. The numbers of quantified telomeres were indicated by "n". Red lines indicate the average length of telomeres. **(G)** Quantification of (E). The percentages of chromosomes with one or more telomere free ends were calculated (n≥ 1000 chromosomes). **(H)** Quantification of (E). The percentages of telomere end-to-end fusion were calculated (n≥ 1000 chromosomes). **(I)** γH2AX foci increase in YTHDC1 depleted U2OS cells. γH2AX and telomeres were detected by anti-γH2AX antibody and C probe respectively. Scale bars, 5 μm. **(J)** Quantification of (I). The mean numbers of γH2AX foci per cell were counted (n≥100 cells × 3 repeats). **(K)** Quantification of (I). The mean numbers of γH2AX foci colocalized with telomeres were counted (n≥100 cells × 3 repeats). **(L)** Western blot analysis of TRF1, TRF2 and POT1 in METTL3 deficient and control U2OS cells. (* P<0.05, ** P<0.01, *** P<0.001).

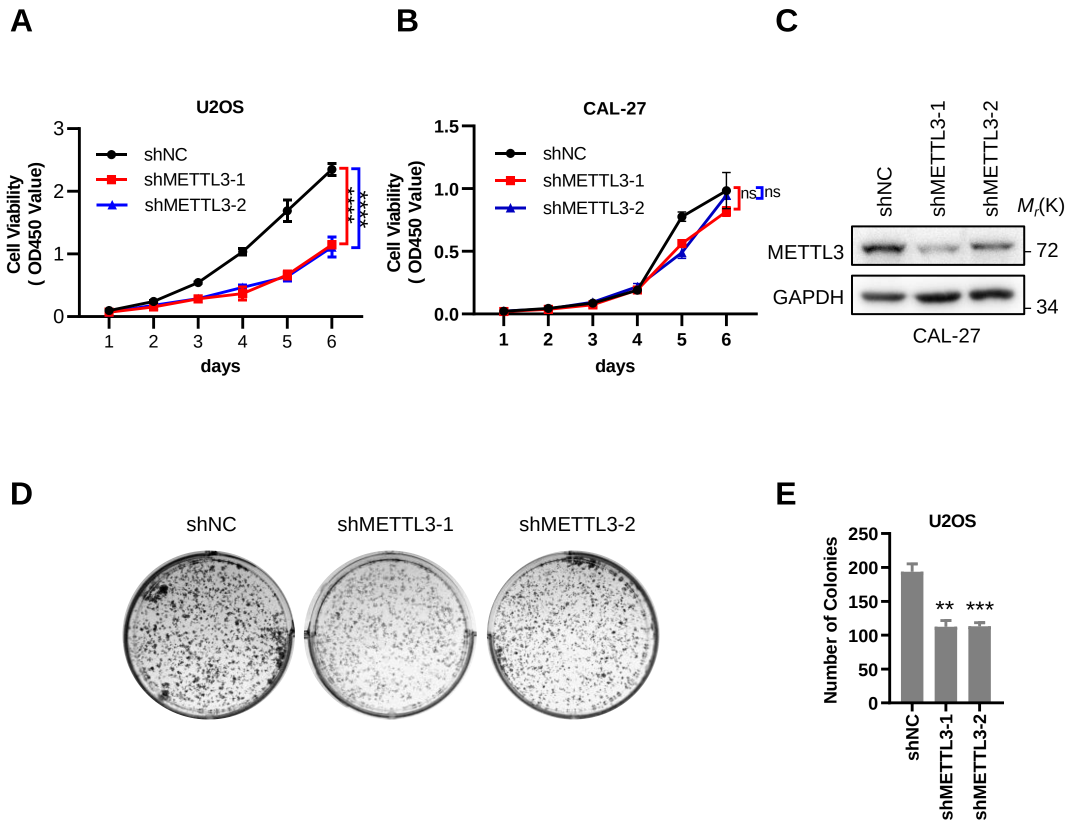

Supplementary Figure S5. METTL3 depletion inhibits ALT cancer cells proliferation. **(A)** Cell viability analysis of METTL3 deficient and control U2OS cells. **(B)** Cell viability analysis of METTL3 deficient and control CAL-27 cells. **(C)** Western blot analysis of METTL3 knockdown efficiency in CAL-27 cells transfected with shRNAs. **(D)** Colony formation assay of METTL3 deficient and control U2OS cells. **(E)** Quantification of (D). The number of colonies was counted. (** P<0.01, *** P<0.001, ****P<0.0001).

Table S1. RT-qPCR primers

primer	Sequence (5'-to -3')
6q-F	TTCTGACGCTGCACTTGAAC
6q-R	TAGTGTGGAAAGCGGGAAAC
7q-F	CTTGGCTGGGGAGAATCT
7q-R	GGTCAGAGAACAGTTAGAAGG
10q-F	GCCTTGCCTTGGGAGAATCT
10q-R	AAAGCGGGAAACGAAAAGC
12p-F	AGTACCACCGAAATCTGT
12p-R	GAGTTGCGTTCTCTTCAG
13q-F	GCACTTGAACCCTGCAATACAG
13q-R	CCTGCGCACCGAGATTCT
15q-F	CAGCGAGATTCTCCCAAGCTAAG
15q-R	AACCCTAACCACATGAGCAACG
19p-F	TTCAGAGTACCACCGAAA
19p-R	GTTCTCCTCAGCACAGAC
ALKBH5-F	CGGCGAAGGCTACACTTACG
ALKBH5-R	CCACCAGCTTTTGGATCACCA
18s RNA-F	ACGGACCAGAGCGAAAGCAT
18s RNA-R	GGACATCTAAGGGCATCACAGAC

Unedited raw images for Figure 2H

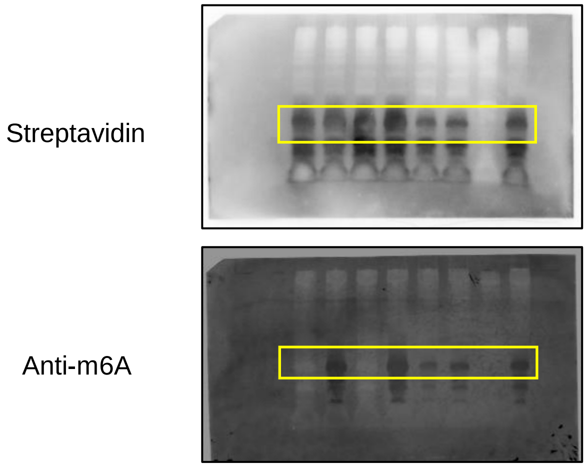

Supplement: gkac1027_Supplemental_File [file gkac1027_supplemental_file.pdf]
